# Synthesis and Characterization of Memantine-Loaded Niosomes for Enhanced Alzheimer’s Disease Targeting

**DOI:** 10.3390/pharmaceutics17020267

**Published:** 2025-02-17

**Authors:** Hasan Turkez, Sena Oner, Ozge Caglar Yıldırım, Mehmet Enes Arslan, Marilisa Pia Dimmito, Çigdem Yuce Kahraman, Lisa Marinelli, Erdal Sonmez, Özlem Kiki, Abdulgani Tatar, Ivana Cacciatore, Antonio Di Stefano, Adil Mardinoglu

**Affiliations:** 1 Department of Medical Biology, Faculty of Medicine, Atatürk University, Erzurum 25100, Turkey; 2 Department of Molecular Biology and Genetics, Faculty of Science, Erzurum Technical University, Erzurum 25100, Turkey; sena.oner66@erzurum.edu.tr (S.O.); ozge.caglar@erzurum.edu.tr (O.C.Y.); enes.aslan@erzurum.edu.tr (M.E.A.); 3 Genescence Biotechnology, Ata Teknokent, Atatürk University Technology Development Zone, Erzurum 25100, Turkey; 4 Department of Pharmacy, University “G. D’Annunzio” of Chieti-Pescara, 6512 Chieti, Italy; marilisa.dimmito@unich.it (M.P.D.); l.marinelli@unich.it (L.M.); ivana.cacciatore@unich.it (I.C.); antonio.distefano@unich.it (A.D.S.); 5 Department of Medical Genetics, Faculty of Medicine, Atatürk University, Erzurum 25100, Turkey; cigdem.kahraman@atauni.edu.tr (Ç.Y.K.); abdulgani@atauni.edu.tr (A.T.); 6 Advanced Materials Research Laboratory, Department of Nanoscience & Nanoengineering, Graduate School of Natural and Applied Sciences, Atatürk University, Erzurum 25100, Turkey; esonmez@atauni.edu.tr; 7 Department of Medical Biochemistry, Faculty of Medicine, Atatürk University, Erzurum 25100, Turkey; ozlem.kiki@saglik.gov.tr; 8 Science for Life Laboratory, KTH-Royal Institute of Technology, SE-17121 Stockholm, Sweden; 9 Centre for Host-Microbiome Interactions, Faculty of Dentistry, Oral & Craniofacial Sciences, King’s College London, London SE1 9RT, UK

**Keywords:** molecular Trojan horse technology, blood–brain barrier, niosomal delivery systems, memantine derivatives, in vitro disease model, Alzheimer’s disease

## Abstract

**Background/Objectives:** Over the past 25 years, numerous biological molecules, like recombinant lysosomal enzymes, neurotrophins, receptors, and therapeutic antibodies, have been tested in clinical trials for neurological diseases. However, achieving significant success in clinical applications has remained elusive. A primary challenge has been the inability of these molecules to traverse the blood–brain barrier (BBB). Recognizing this hurdle, our study aimed to utilize niosomes as delivery vehicles, leveraging the “molecular Trojan horse” technology, to enhance the transport of molecules across the BBB. **Methods:** Previously synthesized memantine derivatives (**MP1–4**) were encapsulated into niosomes for improved BBB permeability, hypothesizing that this approach could minimize peripheral drug toxicity while ensuring targeted brain delivery. Using the human neuroblastoma (SH-SY5Y) cell line differentiated into neuron-like structures with retinoic acid and then exposed to amyloid beta 1–42 peptide, we established an in vitro Alzheimer’s disease (AD) model. In this model, the potential usability of **MP1–4** was assessed through viability tests (MTT) and toxicological response analysis. The niosomes’ particle size and morphological structures were characterized using scanning electron microscopy (SEM), with their loading and release capacities determined via UV spectroscopy. Crucially, the ability of the niosomes to cross the BBB and their potential anti-Alzheimer efficacy were analyzed in an in vitro transwell system with endothelial cells. **Results:** The niosomal formulations demonstrated effective drug encapsulation (encapsulation efficiency: 85.3% ± 2.7%), controlled release (72 h release: 38.5% ± 1.2%), and stable morphology (PDI: 0.22 ± 0.03, zeta potential: −31.4 ± 1.5 mV). Among the derivatives, MP1, **MP2**, and **MP4** exhibited significant neuroprotective effects, enhancing cell viability by approximately 40% (*p* < 0.05) in the presence of Aβ1-42 at a concentration of 47 µg/mL. The niosomal delivery system improved BBB permeability by 2.5-fold compared to free drug derivatives, as confirmed using an in vitro bEnd.3 cell model. **Conclusions:** Memantine-loaded niosomes provide a promising platform for overcoming BBB limitations and enhancing the therapeutic efficacy of Alzheimer’s disease treatments. This study highlights the potential of nanotechnology-based delivery systems in developing targeted therapies for neurodegenerative diseases. Further in vivo studies are warranted to validate these findings and explore clinical applications.

## 1. Introduction

The last few decades represent a pivotal period in understanding the potential of biological molecules used in the treatment of neurological diseases and examining their interaction with the BBB. Especially concerning Alzheimer’s disease (AD), this intricate barrier impedes therapeutic agents from reaching brain tissue, posing a significant challenge in AD treatment [1]. In recent years, researchers have begun to adopt the “molecular Trojan horse” approach, devising strategies to facilitate drugs in crossing the BBB. This method could enable potential treatments to more effectively reach targets within the brain, paving the way for newer and more efficient therapeutic strategies in AD treatment. However, further research is essential for these strategies to achieve clinical success [2]. The “Molecular Trojan Horse” technology stands out as a noteworthy strategy in drug delivery system research. This technology facilitates the more efficient delivery of specifically targeted treatments. When addressing neurological diseases, it offers a particularly promising solution for overcoming the challenges of brain-barrier penetration. The brain-barrier represents a system that only permits a limited number of molecules to enter the brain, complicating drug delivery. The “Molecular Trojan Horse” technology employs nanoparticles or other carrier systems that can traverse this barrier and release therapeutic agents once inside the brain. Consequently, more effective therapeutic strategies can be developed for treating neurological diseases, particularly Parkinson’s and Alzheimer’s. Such treatments have the potential to decelerate the progression of the disease and possibly enhance quality of life [2,3].

In treating AD, memantine serves as an antagonist to NMDA receptors. These receptors are instrumental in mitigating overstimulation and neurotoxic effects through the brain’s glutamatergic signaling. Memantine holds a crucial place in the therapeutic strategies employed, particularly in the advanced stages of AD. Recent studies on memantine derivatives aim to enhance this drug’s efficacy. In fact, Cacciatore et al. (2017) propounded superior chemico-physical properties with good stability in both simulated gastric and intestinal fluids by using novel memantine derivatives **MP1–4** and introduced them as being appropriate for advanced in vivo pharmacokinetic studies [4]. These derivatives could potentially offer more targeted binding capabilities, enabling a more precise approach to addressing the specific neurological damage caused by the condition. Furthermore, evidence suggests that certain derivatives may lower oxidative stress and amplify neuroprotective benefits, presenting additional advantages that surpass the inherent positive effects by memantine [5]. Herein, a previous study it was revealed that novel memantine derivatives involving MEM1–6 exerted prominent neuroprotection against oxidative damage due to their antioxidant portion residues, such as N-acetyl-Cys-OH and N-acetyl-Cys(Allyl)-OH [6]. Hence, attention has been drawn to the potential of these derivatives to mitigate the toxicity of amyloid-beta oligomers, molecules that are significantly involved in the process of neurodegeneration. It is believed that these derivatives could offer avenues for directly addressing the core pathophysiological mechanisms of AD [7]. New studies suggest that memantine derivatives could hold the potential to protect and improve cognitive functions in the brain from the early stages of AD. Moreover, by minimizing the side effects associated with memantine, these derivatives may provide a safer therapeutic alternative for patients. It is anticipated that, in the future, further clinical trials of memantine derivatives will be conducted, potentially leading to a new benchmark in AD treatment. Nonetheless, additional research is required to elucidate the precise mechanisms of these derivatives, and such research is crucial for their future safe and effective application [8].

The BBB serves as a critical diffusion barrier that blocks the entry of substances from the blood into the brain, a necessary function for maintaining the brain’s normal operations and its homeostasis. The BBB is physically formed by tight junctions (TJs), brain microvascular endothelial cells (ECs), neurons, astrocytes, pericytes, and the basal membrane, creating the tightly regulated capillaries within the brain [9,10]. In the endothelial cells of brain capillaries, there are no openings (fenestrations) that permit the diffusion of small molecules and proteins. Connections between endothelial cells create a seamless barrier, heavily limiting the entry of water-soluble compounds into the brain. Surrounding these endothelial cells, pericytes, astrocytes, and the basal membrane contribute to the formation of the BBB, which restricts the movement of substances. Furthermore, brain capillary endothelial cells contain efflux transporters, adding an additional layer of defense against the entry of substances into the brain. The selectivity of the BBB is largely governed by the inter-endothelial connections, through complexes, like adherens junctions, TJs, and gap junctions, controlling its permeability [11]. Previous research has demonstrated the ability of the memantine molecule, which will be utilized in the study, to penetrate the brain. However, the distribution of this molecule in the body results in very low concentrations actually reaching the brain. Increasing the dosage to enhance the brain’s absorption leads to unavoidable toxic effects in tissues and organs. By employing niosomal vesicles for transport, it is possible to achieve both targeted and efficient delivery to the brain while minimizing potential damage to tissues and organs.

Niosomal delivery systems are a key component of nanoparticle-based medication delivery strategies, viewed as an optimistic method for addressing neurological conditions. By utilizing vesicles made from non-ionic surfactants and cholesterol, these systems facilitate the directed delivery of therapeutic agents. Their significance, especially in managing neurodegenerative disorders, stems from their capability to efficiently traverse the BBB and enhance therapeutic levels within precise regions of the brain. In the treatment of neurological diseases, niosomal delivery systems can transport next-generation drugs capable of modulating pathological processes in the brain. These include peptides, antisense oligonucleotides, and RNA-based therapies [12]. According to Patil and colleagues (2015), niosomes are more effective than liposomes in crossing the BBB and delivering a greater amount of medication to brain tissue. These systems are an indispensable tool for optimizing drug delivery strategies in neurological disorders, such as Alzheimer’s disease, Parkinson’s disease, and multiple sclerosis [13]. They can be particularly effective in reducing neuroinflammation and developing neuroprotective strategies. However, further preclinical and clinical research is needed to explore the potential applications of niosomal delivery systems. The full potential of this technology in the treatment of neurological diseases will be better understood with future research [14]. In light of all this information, the improved neuroprotective activity by novel memantine derivatives (**MP1–4**)-loaded niosomes can be considered a substantial strategy for therapeutic design and discovery. This new strategy may simultaneously enhance cell viability and neuroprotection and possess better BBB permeability properties.

## 2. Materials and Methods

### 2.1. Synthesis of Memantine Derivatives (***MP1–4***)

**Figure 1:**
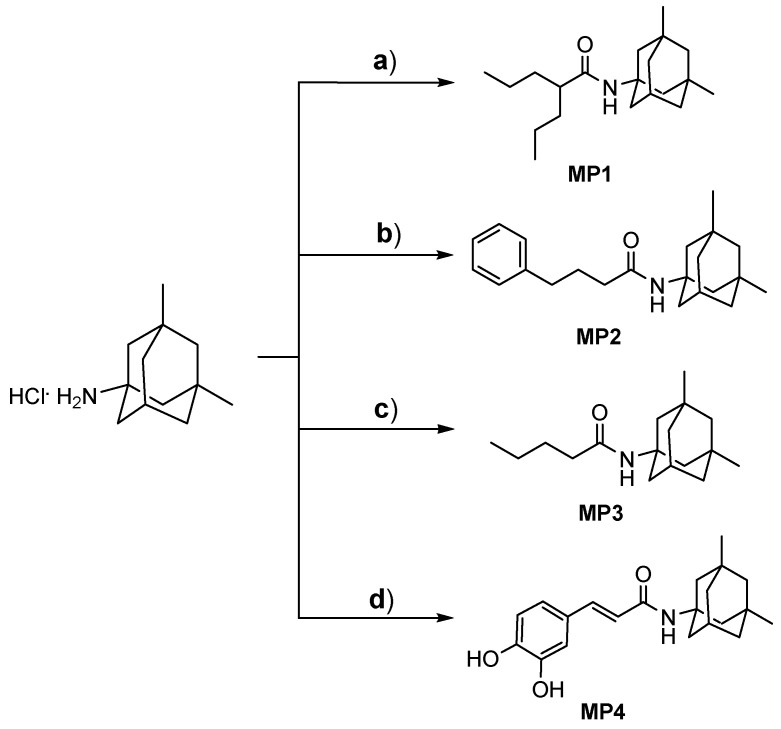
Synthetic strategies to obtain memantine derivatives (**MP1–4**). Reagent and conditions: (**a**) valproic acid, TEA, and ethylchloroformate in dry THF/DMF, 3 h, rt; (**b**) phenylbutyric acid, TEA, and ethylchloroformate in dry THF/DMF, 3 h, rt; (**c**) butyric acid, TEA, and ethylchloroformate in dry THF/DMF, 3 h, rt; (**d**) caffeic acid, TEA, HOBt, and DCC in dry DMF, 15 h, rt.

In our previous study, **MP1–4** were designed and synthetized by employing the synthetic methods outlined in Figure 1 [4,6]. Briefly, ethylchloroformate was used as a reagent for amino group activation in amide linkage synthesis between memantine and the suitable carboxylic derivatives. **MP1–4** were obtained in good yields (>50%, Supplementary Data S1).

### 2.2. Human Fibroblast Cell Cultures and Micronucleus Analysis

The human fibroblast cell line (ATCC^®^ PCS-201-012™ Manassas, VA, USA) was propagated in DMEM culture medium enriched with a 1% mixture of penicillin/streptomycin antibiotics and 10% fetal bovine serum (FBS) and maintained in an atmosphere containing 5% CO_2_ at a temperature of 37 °C until they reached full confluence. To identify nuclei alterations, Hoechst 33258 fluorescent dye was employed. In this procedure, fibroblast cells underwent a 24 h treatment with various concentrations of drug formulations (ranging from 0 to 500 µg/mL, specifically at 8.62 µg/mL, 16.13 µg/mL, 31.25 µg/mL, 62.5 µg/mL, 125 µg/mL, 250 µg/mL, and 500 µg/mL) determined by the IC_50_ value for synthesized compounds. Following the treatment period, the cells were stabilized using 4% paraformaldehyde at a cool temperature of 4 °C for 30 min. The cells were then rinsed with PBS and exposed to 1 µM of the Hoechst 33258 dye for a brief duration of 5 min [15]. After the staining process, the cells were examined and imaged using a Leica^®^ DM IL LED (Leica Microsystems, Wetzlar, DE) fluorescent microscope.

### 2.3. Synthesis of Niosomal Delivery Systems

The niosomes were prepared by the thin film hydration method in a round-bottom flask with some minor modifications, as previously reported [16]. The chloroform was evaporated at 110 rpm, 60 °C for 1 h using a rotary evaporator (Hei Dolph Instruments, Schwabach, DE). Thin films were hydrated with **MP1–4** (1 mg/mL) in PBS at 60 °C, 120 rpm for 30 min. The samples were then stored in a refrigerator at 4 °C until the next experiment was performed. The compounds used in the synthesis of niosomes are listed in Table 1.

### 2.4. Characterization and Size Distribution of Loaded Niosomes

Single-use polystyrene cuvettes were utilized to assess the particle size distribution and to calculate the median particle size (Z-average). The median size was determined at a temperature of 25 °C by employing a Zetasizer from Malvern Instruments Ltd., Malvern, UK. Each size measurement was conducted in triplicate and the results were presented as the average ± standard deviation. The examination of the niosomes’ structure was performed with the aid of scanning electron microscopy (FE-SEM) using the Quanta FEG 250 model, (FEI, Eindhoven, The Netherlands). For the SEM analysis, 50 μL of the PBS-dissolved niosomes solution was deposited onto a sterile glass slide. After drying, the sample was sputter-coated with a thin gold layer, approximately 100 Å thick, for 4 min to enhance the conductivity. The gold-coated sample was then analyzed using SEM at an accelerating voltage of 15 kV to observe its morphology.

### 2.5. Drug Loading Analyses of Niosomes

The efficiency of **MP1–4** encapsulation within the niosomes was evaluated by quantifying the unencapsulated **MP1–4**. This was achieved by centrifuging the niosomal mixtures at 4 °C for 20 min at a speed of 4000 rpm, utilizing an Amicon Ultra centrifuge filter unit (Millipore Merck, Darmstadt, DE). The optical density of the supernatant containing free **MP1–4** was measured at a wavelength of 236 nm with a UV–Vis spectrophotometer (Eppendorf^®^ 580R centrifuge, Hamburg, DE). By determining the concentration of the free drug in the supernatant, we were able to calculate the unknown amount of encapsulated drug. The encapsulation efficiency percentage was then computed based on the initial molecule concentration (C_i_) used in the niosome preparation versus the concentration found post-centrifugation (C_f_) [17], as in the following equation:

% Loading capacity = [(C_i_ − C_f_)/C_i_] × 100 (1)

### 2.6. Drug Release Analysis

To investigate the drug’s release profile from the niosomes, an in vitro release study was performed. For the release study, we used loading rates calculated by a drug loading study. A 2 mL aliquot of the optimized niosomal formulation was enclosed within a dialysis membrane (with a 12 kDa molecular weight cut-off) and incubated for 72 h at 37 °C. This incubation occurred in a stirring environment within 50 mL of a release solution composed of phosphate-buffered saline with 0.5% sodium dodecyl sulfate (PBS-SDS) by *w*/*v*. Samples of 1 mL were systematically withdrawn at specific intervals (1, 2, 4, 8, and 24 h) from the release medium, with each withdrawal immediately compensated for by an equivalent volume of fresh release medium. The optical density of these samples was measured at 236 nm using a spectrophotometer. From these measurements, the quantity of memantine released was calculated, and the release percentages were reported as a proportion of the total drug release.

### 2.7. SH-SY5Y Cell Cultures and Differentiation with Retinoic Acid

The SH-SY5Y neuroblastoma cell line, frequently used as an in vitro model of Alzheimer’s disease, has been cultured to transform into mature neuron-like structures through differentiation with retinoic acid (RA). For this purpose, the SH-SY5Y neuroblastoma cell line was grown in a DMEM:F12 nutrient medium environment with 10% fetal bovine serum (FBS) growth factor, 1% Penicillin/streptomycin, and 5% CO_2_ at 37 °C. The nutrient medium was refreshed every three days, with half the volume changed. Cells were subcultured after treatment with trypsin/EDTA for 5 min. To differentiate SH-SY5Y cells, the medium containing 10 µM RA (DMEM:F12 + 15% FBS) was added. Five days after the addition of RA, the cells were washed three times with PBS and incubated in DMEM:F12. The differentiation of the cells was determined by cell cycle analysis using a flow cytometry system. Then, a wide concentration range of the Aβ1-42 molecule (8.62 µg/mL, 16.13 µg/mL, 31.25 µg/mL, 62.5 µg/mL, 125 µg/mL, 250 µg/mL, and 500 µg/mL) was added to the cell culture to calculate the dose that kills 50% of the cells (IC_50_). Drug formulations prepared in the concentration range of 0–500 µg/mL (8.62 µg/mL, 16.13 µg/mL, 31.25 µg/mL, 62.5 µg/mL, 125 µg/mL, 250 µg/mL, and 500 µg/mL) were applied in triplicate to cell cultures that had completed cellular differentiation and were treated with the IC_50_ concentration of Aβ1-42 [18]. Converted cell cultures were used as a negative control, and cell cultures with only amyloid beta were used as a positive control without adding the synthesized molecules. The viability rates in the in vitro *AD* model that we intended to create were analyzed with MTT cytotoxicity tests.

### 2.8. MTT (3-(4,5-Dimethylthiazol-2-yl)-2,5-Diphenyltetrazolium Bromide) Cell Viability Investigations

Differentiated SH-SY5Y cells were seeded into 96-well plates, with each well containing 100 μL of culture medium with 2 × 10^4^ cells. After the cells adhered, drug formulations prepared in the concentration range of 0–500 µg/mL (8.62 µg/mL, 16.13 µg/mL, 31.25 µg/mL, 62.5 µg/mL, 125 µg/mL, 250 µg/mL, and 500 µg/mL) at the IC_50_ concentration of Aβ1-42 were applied to the cells and incubated for 24 h. At the end of the incubation period, 10 μL of MTT mixture was added to each well for MTT analysis. The mixture was gently stirred using a circular mixer for 1 min and then incubated in a CO_2_ incubator at 37 °C for 3–4 h. After incubation, dark crystals of formazan were observed at the bottom of the wells. The culture medium was carefully aspirated from each compartment without disturbing the cell layer. Alternatively, before aspiration, the plate was centrifuged at 400× g for 10 min to settle non-adherent cells. Then, 100 μL of crystal solvent solution (DMSO) was added to each compartment, and the absorbance of each sample was measured at 570 nm using a microplate reader [18].

### 2.9. In Vitro Blood–Brain Barrier (BBB) Permeability Analyses

**Figure 2:**
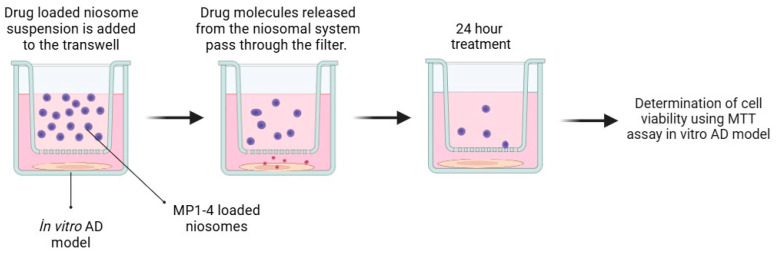
Schematic diagram summarizing in vitro BBB permeability analyses.

In vitro BBB models were created using a transwell culture system for BBB permeability analysis. Initially, bEnd.3 cells were seeded inside the membrane of the plate at a starting density of 1 × 10^5^ cells/well and cultured for about a week in DMEM growth medium supplemented with 10% fetal bovine serum. Subsequently, the upper compartments containing bEnd.3 monolayers were transferred to plates containing SH-SY5Y cells coated at the bottom of the lower compartments. Then, carrier particles were added to the apical upper chambers of the BBB models at a final concentration of 0.1 mg/mL. Approximately 24 h after incubation, viability analyses were conducted on the SH-SY5Y disease model formed in the lower layer to determine BBB permeability capacities [19]. A schematic diagram of the method is shown below (Figure 2). Prior to starting the experimental procedures, the bEnd.3 cells were cultured until 90–100% confluency was achieved, as this confluency level is necessary for tight junction formation and optimal barrier properties. The integrity of the cell monolayer was then evaluated under an inverted microscope to confirm confluence and uniform cell distribution, ensuring the reliability of the in vitro BBB model.

### 2.10. Statistical Analyses

Group analyses were performed with the one-way ANOVA procedure. Studies were compared with Dunnett’s test (against a control) and by using Tukey’s multiple comparison analyses. Each experiment was performed in triple replicates. The level of significance was set at 5% (*p* < 0.05). Calculations were performed using the GraphPad Prism software, version 7.0.

## 3. Results

### 3.1. Cytotoxicity and Genotoxicity Analyses of ***MP1–4*** on Human Dermal Fibroblast (HDFa) Cell Cultures

**Figure 3:**
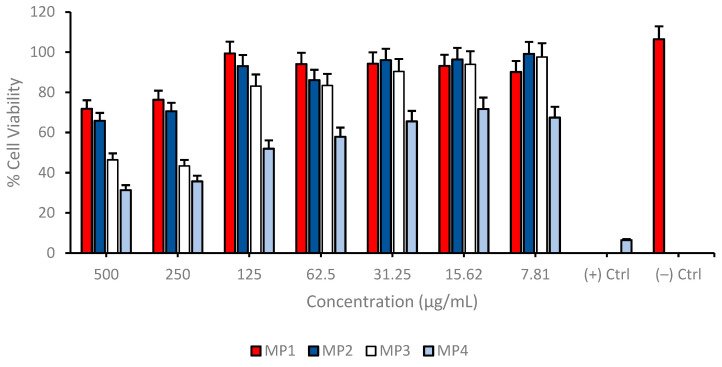
Determination of cytotoxic properties of synthesized memantine derivatives (**MP1–4**) in cultured human fibroblast cells (HDFa) using MTT cell viability assay. Group analyses were performed with the one-way ANOVA procedure, and comparisons were made using Dunnett’s test (against the control).

The human fibroblast cell line HDFa was cultured and tested for toxicity with 4 different memantine derivatives in 48-well plates. The experiments, conducted in triplicate, included both treated and untreated (negative control) cell cultures. Post-treatment, the cells were incubated with an MTT mixture to assess cell viability, followed by the addition of a crystal solvent solution (DMSO) to dissolve formazan crystals formed in viable cells. The absorbance of each sample was measured to evaluate the cytotoxic effects of the memantine derivatives on the fibroblast cells. The synthesized memantine derivatives **MP1–3** have been demonstrated to cause no significant cytotoxic effects at concentrations below 125 µg/mL (Figure 3).

**Figure 4:**
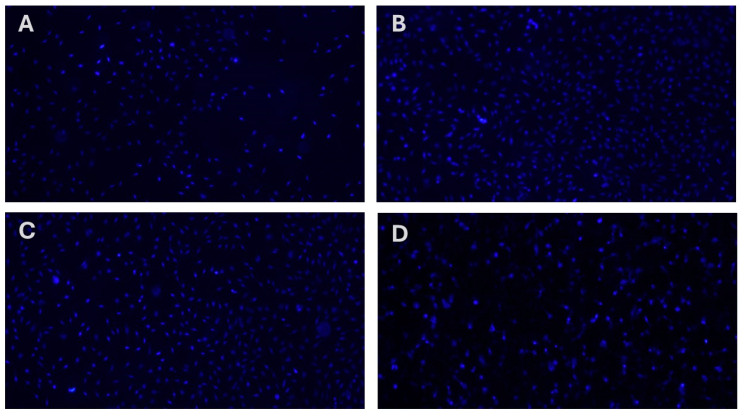
Analysis of the genotoxic properties of synthesized memantine derivatives (**MP1–4**) in cultured human fibroblast cells (HDFa) using Hoechst 33258 fluorescent staining of cell nuclei. (**A**) **MP1**, (**B**) **MP2**, (**C**), **MP3** and (**D**) **MP4**.

Only compound **MP4** exhibited a significant cytotoxicity at each concentration and decreased cell viability nearly 40% for each application. This observation suggests that these compounds are relatively non-toxic to cells, which is a crucial consideration in the development of new therapeutic agents, as it indicates a lower risk of adverse effects on healthy cells. Furthermore, the analysis of cellular nuclei using Hoechst 33258 fluorescent dye has revealed that **MP1–4** do not lead to significant genotoxic effects (Figure 4 and Table 2). Genotoxicity refers to the ability of substances to damage genetic information in cells, leading to mutations that could potentially cause cancer or other genetic disorders. The absence of significant genotoxic effects in this study suggests that **MP1–3** are not only non-toxic at certain concentrations but also do not pose a significant risk of causing genetic damage. This combination of low cytotoxicity and minimal genotoxicity is particularly promising for the development of new drugs, as it suggests that these compounds could be safer alternatives to existing treatments. The findings highlight the potential of **MP1–3** for further research and development, especially in applications where minimizing the risk to healthy cells is paramount. This could open up new avenues for the use of **MP1–3** in medical treatments, making them candidates for more in-depth studies to explore their efficacy and safety profiles in a broader range of applications.

### 3.2. Characterization Analyses of Niosomes

**Figure 5:**
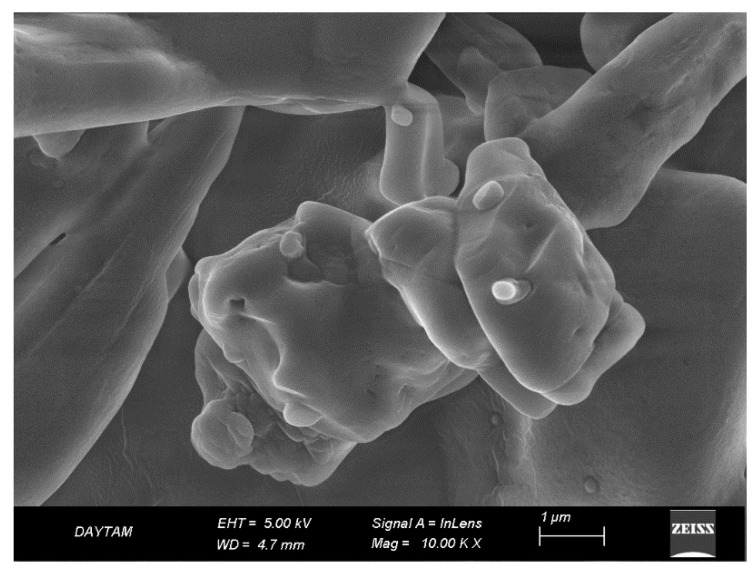
Scanning electron microscope (SEM) image showing the surface topography and morphology of niosomes prepared by the thin film method.

The detailed analysis conducted using an electron microscope revealed that the synthesized niosomes exhibit a homogeneous distribution, containing particles with similar and regular structures closely sized to each other. Size analysis indicates that the niosomes have a size distribution ranging approximately from 170 to 300 nm. The SEM images showed that the niosomes have a spherical structure. This suggests that the particle sizes are distributed closely within this range, indicating the physical stability of the product and its suitability for potential biological applications. This size range could also be considered as an indicator that niosomes could be used as carrier systems for specific cell lines. Homogeneous distribution is a desired characteristic, especially in certain applications, like controlled drug release, as it can ensure a more uniform uptake of the drug by the targeted cells or tissues (Figure 5).

With zeta potential analysis, the surface charge and polydispersity index (PDI) of the prepared niosomal delivery system and drug-loaded delivery systems were calculated. The surface charge of the empty delivery system was determined to be −14.4, with a PDI value of 0.302. These values indicate that the prepared system falls within a stable range in terms of energy and PDI. Loading the delivery system with four different drugs altered the surface energy of the transport system to −20.8 mV, −19.7 mV, −22.6 mV, and −21.5 mV for drugs 1–4, respectively. Furthermore, the varying surface energy and PDI values were within the range required for drug-loaded delivery systems, especially those targeting neurodegenerative diseases (Table 3).

The loading capacities of the synthesized memantine derivative compounds in the niosomal drug delivery system were examined in detail. In this study, loading efficiency was determined by centrifuging niosomal formulations using an Amicon Ultra centrifuge filter device (Millipore Merck, Burlington, MA, USA) at 4000 rpm for 20 min at 4 °C. The amount of free drug remaining was recorded using a UV–Vis spectrophotometer (Eppendorf^®^ 580R centrifuge, Hamburg, DE). As a result, it was found that **MP1**, **MP3**, and **MP4** could be loaded onto niosomes at rates of 85% or higher. However, the loading capacity of the memantine derivative **MP2** was determined to be 53%, which is below the desired capacity. These results demonstrate the loading capacities of the compounds into the niosomal drug delivery system, indicating that the loading capacity of the memantine derivative **MP2** is lower compared to the other compounds (Table 3).

**Figure 6:**
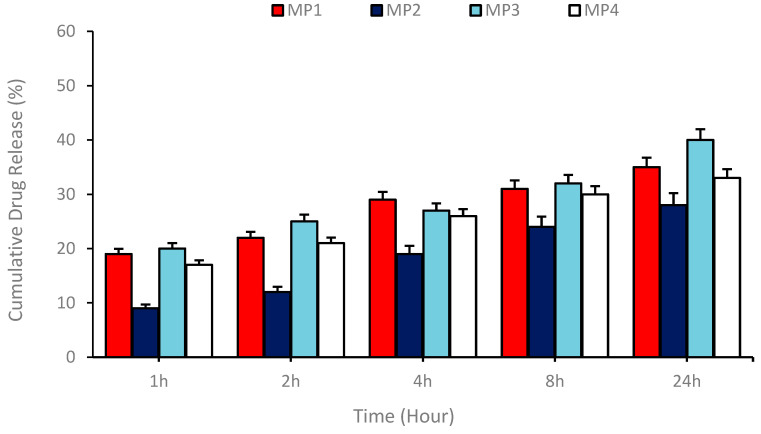
Drug release profile showing the release of **MP1–4** drugs from niosomal carriers prepared by the thin film hydration technique at certain time intervals for 24 h.

Drug release capacities with **MP1–4** were determined using the niosomal release systems generated. These systems exhibited slow and proportional drug release observed by sampling at certain time intervals during in vitro drug release experiments. This feature offers an important advantage in terms of controlled drug release. The drug release study was carried out by measuring the absorbance using a spectrophotometer on samples taken at specific time intervals (1, 2, 4, 8, and 24 h). These measurements were carried out at the max peak of the drugs, and the data obtained were used to determine the amount of memantine derivative drug released. The results present the percentages of the released content as a percentage of total release. As a result, it was found that the most optimal drug release occurred with the memantine **MP1** compound. This finding suggests that the memantine **MP1** compound can be effectively utilized as a potential therapeutic agent in drug delivery systems. It was also concluded that a slow and proportional release occurred over the indicated time period, indicating the potential for controlled drug release (Figure 6).

As demonstrated in Figure 6, the release kinetics were found to be consistent with various mathematical models, including zero order, first order, and Higuchi models. The calculations indicated that the prepared drug-loaded niosomal transport system exhibited optimal compatibility with the Higuchi model. The Higuchi coefficients (*kH*) for **MP1–4** were found to be 10.36, 7.55, 12.17, and 10.82, respectively, thereby indicating that the release process is governed by a diffusion-controlled mechanism and that the drug is released from the matrix over time by means of a concentration gradient.

### 3.3. Anti-Alzheimer’s Disease Potential of the Synthesized Compounds

**Figure 7:**
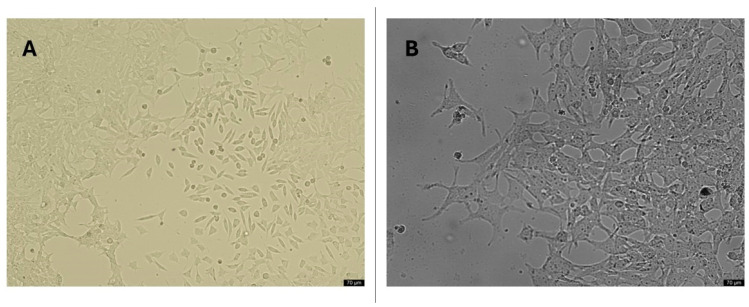
Morphological cell structures of differentiated SH-SY5Y cells. (**A**) 20× resolution of undifferentiated cell cultures; (**B**) 20× resolution of differentiated cell cultures (application of 10 µM all-trans RA to cell culture for 11 days).

An in vitro disease model was constituted by transforming the SH-SY5Y neuroblastoma cell line with retinoic acid (RA) into mature neuron-like structures, which is frequently used as an in vitro model of AD. Cell differentiation was determined by flow cytometry analysis of the cell cycle analyses. Following the differentiation process, noticeable changes in cell morphology were observed, transitioning from cuboidal shapes to slender, body-like structures. Distinct axonal and dendritic formations emerged, accompanied by evident cell-to-cell connections indicative of cellular interactions, as visualized under an inverted microscope (Figure 7).

To confirm successful differentiation, flow cytometry was utilized to analyze the cell cycle distribution within the cultures. The findings revealed a significant shift in cell populations from the S phase to the G1 phase, with a concurrent decrease in G2 phase populations (Table 4). These results suggest a reduction in DNA synthesis and cell division, characteristic of mature neurons.

**Figure 8:**
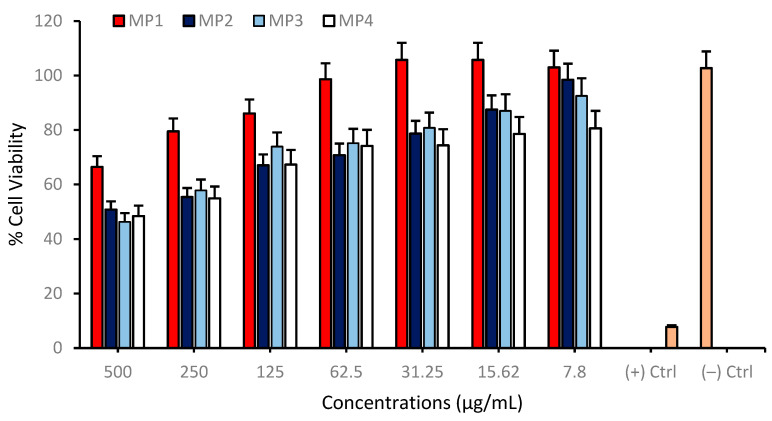
Determination of the cytotoxic properties of **MP1–4** in the differentiated SH-SY5Y cell cultures resembling mature neurons using the MTT cell viability test. Group analyses were performed with the one-way ANOVA procedure, and comparisons were made using Dunnett’s test (against the control).

Cell viability was assessed across a range of concentrations from 7.81 to 500 µg/mL for each compound on the differentiated SH-SY5Y cell cultures to investigate possible cytotoxic properties of **MP1–4**. The results indicate a concentration-dependent decrease in cell viability for all compounds tested. At the highest concentration of 500 µg/mL, the cell viability for **MP3** and **MP4** was significantly reduced, suggesting a higher cytotoxic effect at this concentration. In contrast, **MP1** and **MP2** showed relatively higher cell viability at the same concentration, indicating lower cytotoxicity or a more favorable cell tolerance at this dosage. As the concentration of the compounds decreased, there was a general trend of increasing cell viability, with the lowest concentration of 7.81 µg/mL showing the highest viability across all compounds. This suggests that the cytotoxic effects of the compounds are diminished at lower concentrations (Figure 8). Comparatively, **MP1** maintained the highest cell viability at higher concentrations, followed closely by **MP4**, while **MP2** and **MP3** exhibited a more pronounced decrease in cell viability at the same concentrations. Also, the IC_50_ values for **MP1–4**, presented in Table 5, demonstrate variations in their concentration-dependent effects on cell viability in the differentiated SH-SY5Y cell culture, although all compounds exhibited relatively low cytotoxicity within the tested concentration range. **MP1** showed the highest IC_50_ value (5618.61 µg/mL), indicating the lowest impact on cell viability, while **MP2** displayed a lower IC_50_ value (456.79 µg/mL), suggesting a more pronounced effect at higher concentrations. **MP3** and **MP4** had intermediate IC_50_ values of 576.40 µg/mL and 658.80 µg/mL, respectively.

**Figure 9:**
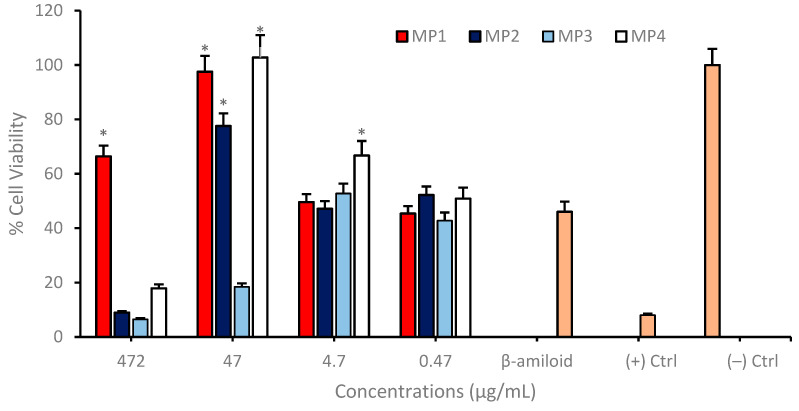
Determination of the neuroprotective properties of **MP1–4** in an experimental AD model. Group analyses were performed with the one-way ANOVA procedure, and comparisons were made using Dunnett’s test (against the amyloid beta application). The level of significance was set at 5% (*p* < 0.05). Significant differences compared to the control group are indicated by an asterisk (*).

**MP1–4** were further evaluated for their neuroprotective properties using differentiated SH-SY5Y neuroblastoma cell cultures, which were exposed to a 10 µM concentration of amyloid beta 1−42 peptide, corresponding to the IC_50_ value. This peptide is instrumental in AD pathology and serves as a pertinent stressor for assessing the neuroprotective efficacy of potential therapeutic agents. In these experiments, compounds **MP1**, **MP2**, and **MP4** demonstrated a pronounced neuroprotective effect against the deleterious impact of amyloid beta. Notably, at a concentration of 47 µg/mL, these compounds significantly enhanced cell viability when compared to the control group, which was treated solely with amyloid beta. This elevation in cell viability suggests that **MP1**, **MP2**, and **MP4** may interact with cellular pathways in a manner that mitigates the toxic influence of amyloid beta, or they may facilitate cellular mechanisms that promote survival in the presence of amyloid beta. However, the memantine derivative **MP3** did not exhibit significant cell viability enhancement up to 472 µg/mL concentration (Figure 9).

**Figure 10:**
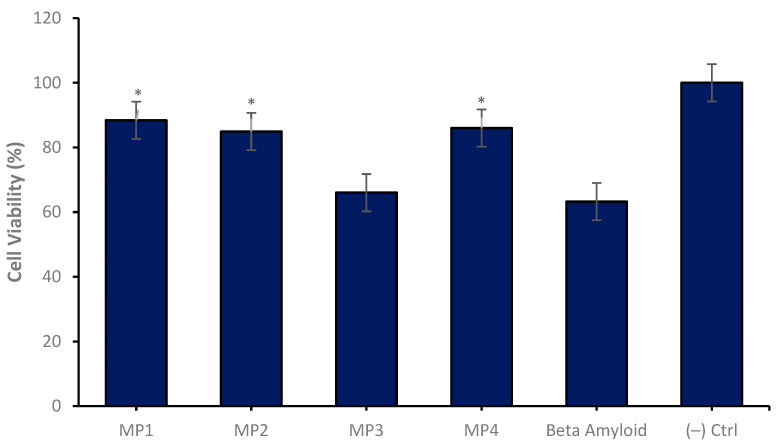
Neuroprotective properties of the **MP1–4** (47 µg/mL) on the experimental AD model for 24 h of application. Group analyses were performed with the one-way ANOVA procedure, and comparisons were made using Dunnett’s test (against the amyloid beta application). The level of significance was set at 5% (*p* < 0.05). Significant differences compared to the control group are indicated by an asterisk (*).

In the conducted in vitro studies, the neuroprotective effects of memantine derivatives encapsulated within a nanosomal drug delivery system were evaluated using the SH-SY5Y neuroblastoma cell line as a model for AD. The differentiated neuronal cultures were treated with memantine derivatives which were loaded into the niosomal drug delivery systems at a uniform concentration of 47 µg/mL across three independent experimental repeats. The outcomes demonstrated that certain memantine derivatives, most notably **MP1**, **MP2** and **MP4**, conferred a significant degree of neuroprotection when compared to control cultures treated solely with amyloid beta. Notably, these derivatives augmented cell viability by approximately 40%, highlighting their potential utility as therapeutic agents (Figure 10).

**Figure 11:**
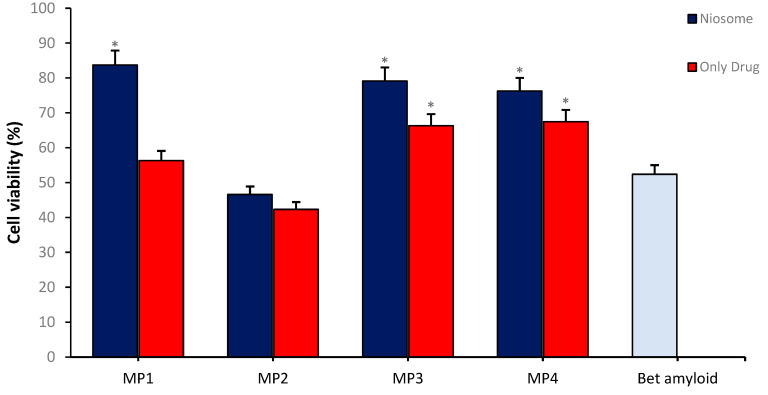
The neuroprotective effect of MP1–4 and the drug/carrier system on cell viability in the Alzheimer’s disease model using an in vitro BBB permeability test. Group analyses were performed with the one-way ANOVA procedure, and comparisons were made using Dunnett’s test (against the amyloid beta application). The level of significance was set at 5% (*p* < 0.05). Significant differences compared to the control group are indicated by an asterisk (*).

In vitro BBB permeability analyses were conducted by establishing in vitro transwell BBB models, within which the effects of drug-loaded **MP1–4** were studied. During the analytical process, bEnd.3 cells were cultured in a transwell system at an initial density of 1 × 10^5^ cells/well for one week, after which they were co-cultured with SH-SY5Y cells at the bottom of the cell culture plate. Carrier particles at a final concentration of 0.1 mg/mL loaded with 47 µg/mL of candidate compounds) and non-loaded memantine derivatives (47 µg/mL concentration) were added to this integrated system, followed by a 24 h incubation period, and subsequent viability analyses were performed on the SH-SY5Y disease model. The results indicate that the memantine derivatives **MP1**, **MP3**, and **MP4** loaded into the transport system exhibited a higher effect on cell viability and a significant increase in neuroprotective properties compared to non-loaded drug derivatives. These findings suggest that the transport system plays a supportive role in enhancing the ability of the drug derivatives to cross the BBB, thus indicating that the transport system could offer significant advantages as a potential therapeutic method (Figure 11).

## 4. Discussion

In this study, the transport and release of **MP1–4** through niosomal delivery systems have been examined. The developed system indicates a great potential for **MP1–4** in terms of controlled drug release and the efficiency of the transportation process. Our findings demonstrate that **MP1–4** can be loaded with high efficiency using niosomal delivery systems. Similar niosomal delivery systems in the literature have been described as a tool to optimize drug release and enable drugs to reach their target receptors more effectively [20]. In particular, analyses conducted with UV–Vis absorption spectra have confirmed that memantine derivatives are loaded in a stable manner and exhibit a controlled drug release profile. Controlled drug release is critically important in terms of enhancing the therapeutic index and reducing side effects. Drug delivery systems, particularly niosomal systems, have the potential to enhance the bioavailability of drugs and optimize their therapeutic efficacy. Memantine is a prominent drug used in the treatment of AD, and this study demonstrates that memantine derivatives **MP1–4** can be transported using niosomal delivery systems. This finding has the potential to further improve current treatment methods [21]. Studies mentioned in the literature demonstrate that niosomal delivery systems are an important tool in terms of the efficiency of the transportation process and the optimization of drug release profiles [22].

Multiplexed memantine derivatives, one of the key components in the treatment of AD, have been the focus of many studies in recent years. Our current study has evaluated the neuroprotective effects of memantine **MP1–4** derivatives in an in vitro AD model. Our findings have shown that the use of niosomal delivery systems can significantly increase cell viability with **MP1–4**, which highlights their therapeutic potential. The differentiated neuronal cultures were treated with memantine derivatives loaded into the niosomal drug delivery system at a uniform concentration of 47 μg/mL across three independent experimental repeats. Among the tested derivatives, **MP1** and **MP2** demonstrated significant neuroprotective effects within their effective concentration ranges, with **MP1** showing optimal activity at 15.6–62.5 μg/mL and **MP2** at 31.25–125 μg/mL. Conversely, **MP4** exhibited cytotoxic effects at concentrations as low as 31.25 μg/mL, indicating a narrower therapeutic window. Despite these limitations, **MP1** and **MP2** augmented cell viability by approximately 40%, highlighting their potential utility as therapeutic agents for Alzheimer’s disease. Recent studies have revealed that nanotechnology-based drug delivery systems could contribute to significant advancements in the treatment of AD [23,24]. In particular, niosomal delivery systems can enhance the neuron-protective capabilities by enabling drugs to reach the brain more effectively [25]. From this perspective, the transportation of memantine derivatives through such a system could create new opportunities for protecting neurons and alleviating the symptoms of AD. This study represents an important step in the drug development process and highlights memantine derivatives as potential new therapeutic agents for the treatment of AD. While the current study provides promising insights into the neuroprotective effects of memantine derivatives encapsulated in a niosomal drug delivery system, further research is essential to translate these findings into clinical relevance. Specifically, evaluating the efficacy of **MP1–MP4** in in vivo models is critical to understanding their pharmacokinetic and pharmacodynamic profiles in a more complex biological environment. In vivo studies can provide valuable information on the compounds’ ability to cross the BBB, their distribution in target tissues, and their long-term safety and toxicity. Additionally, these models can help elucidate potential side effects, interactions with other biological systems, and mechanisms of action under physiological conditions. Advanced analyses, such as molecular imaging and behavioral assessments in Alzheimer’s disease models, can further validate the therapeutic potential of **MP1–MP4**. These investigations will not only assess their suitability for clinical applications but also help optimize formulation parameters, such as drug release profiles and dosing regimens, to enhance their efficacy and safety in humans. Ultimately, the combination of in vitro and in vivo studies is indispensable for advancing these compounds toward clinical translation [26].

The size of nanoparticles is an essential parameter when considering the BBB. Crossing the BBB represents a significant obstacle in the treatment of nervous system diseases. This study tested the hypothesis that the niosomal delivery system could enhance the ability of memantine derivatives to cross the BBB. The results have revealed that niosomal delivery systems can transport memantine derivatives across the BBB to reach target cells. Previous studies have indicated that particles measuring below 100 nm are particularly well-suited for the desired size, while particles ranging from 100 to 200 nm are deemed suitable for this target. Zeta potential analysis is a method used to determine the surface charge of particles. In particular, studies have reported that the range of −30 to +30 mV indicates stable systems. The enhancement of surface charge exerts a direct influence on stability, thereby affecting the repulsion force exerted by particles on one another. Previous research on BBB permeability also indicates that nanotechnology-based delivery systems can be effective in overcoming such barriers. For example, a previous study has shown that nanoparticles can cross the BBB and potentially be used in treating central nervous system diseases [2]. Similarly, it has been observed that niosomal delivery systems can enhance the BBB permeability of compounds, like memantine derivatives. These findings are consistent with a previous study, which highlighted the potential of niosomal delivery systems in the treatment of nervous system diseases [27]. Since memantine is an agent approved by the FDA for the treatment of AD, enhancing the capacity of its derivatives to cross the BBB could represent a significant advancement in the treatment of the disease [28,29].

Previous studies have highlighted the utility of the bEnd.3 cell line in establishing in vitro transwell models of the BBB for evaluating permeability, transport mechanisms, and barrier integrity. In a previous study, this model was optimized by culturing bEnd.3 cells on transwell inserts coated with basement membrane substitutes, like fibronectin and collagen, and then assessing permeability with Lucifer Yellow. Similarly, Aday et al. developed a protocol using gelatin-coated transwells to form tight junctions, enabling nanoparticle permeability studies. Further refined BBB modeling was demonstrated by investigating the effects of extracellular matrix components, such as Matrigel, on barrier functionality. These studies collectively demonstrate the versatility of the bEnd.3 transwell system as a reproducible and adaptable tool for BBB research, providing valuable insights into drug delivery, neurotoxicity, and barrier physiology [30,31,32]. It can thus be concluded that the **MP1–4** loaded transport systems prepared in this study are stable within this context. This is evidenced by the regular distribution of repulsive forces exerted by the particles. Consequently, the therapeutic efficacy of the drug on the target can be augmented.

In this study, we explored the potential of combining niosomal delivery systems with **MP1–4** in the treatment of AD. The role of drug delivery systems in the treatment of AD has been emphasized by various studies, and advancements in this area hold a significant place in developing new and effective approaches to the treatment of the disease. In this context, when **MP1–4** are combined with niosomal delivery systems, an increased capacity to cross the BBB and a more effective reach to the target cells have been observed. Overcoming the BBB presents a significant challenge in the treatment of neurodegenerative diseases, and the capacity of niosomal delivery systems can significantly enhance the effectiveness of the treatment [33]. Additionally, it has been determined that memantine **MP1–4** derivatives can significantly enhance cell viability, demonstrating the therapeutic potential of these compounds. This is consistent with similar studies in the literature; for instance, it has been reported that memantine derivatives can provide neuroprotective effects in in vitro models [34]. Another advantage of combining memantine derivatives with niosomal systems is that these systems can provide controlled drug release, thereby enhancing the therapeutic efficacy of the drugs [35]. Previous studies on memantine and its derivatives have shown that these compounds could have neuroprotective effects in AD, but crossing the BBB remains an obstacle [36]. This study demonstrates that niosomal systems could facilitate the crossing of the BBB by memantine derivatives, thereby making the targeted treatment more effective.

## 5. Conclusions

This study highlights the promising potential of niosomal delivery systems for the transport and release of memantine derivatives (**MP1–4**) in the treatment of Alzheimer’s disease (AD). Our findings demonstrate that these systems enable high-efficiency loading and controlled release of **MP1–4**, which is critical for optimizing therapeutic outcomes while minimizing side effects. Importantly, niosomal delivery systems have shown the capability to enhance the permeability of **MP1–4** across the BBB, a major hurdle in treating central nervous system disorders. This advancement not only facilitates effective drug delivery to target cells but also underscores the neuroprotective effects of memantine derivatives in in vitro AD models, as evidenced by increased cell viability. The integration of nanotechnology into drug delivery, particularly through niosomal systems, represents a significant step forward in overcoming challenges, like BBB permeability and achieving targeted treatment. By combining memantine derivatives with niosomal carriers, this study opens new avenues for improving the treatment of AD, offering enhanced bioavailability, controlled drug release, and better therapeutic efficacy. While these findings are encouraging, further research is essential to validate the efficacy of **MP1–4** in in vivo models and assess their potential for clinical applications. Future studies should also explore advanced delivery systems and their scalability for broader therapeutic use. Ultimately, this research contributes to the development of innovative treatment strategies for AD and lays the foundation for future advancements in neurodegenerative disease therapies.

## Figures and Tables

**Table 1:** Composition of niosomal formulation.

Formulations	Surfactant	Drug	Lipid–Drug Molar Ratio	Drug Concentration (mg/mL)	Surfactant: Cholesterol Molar Ratio
N1	Span60	MP1	2	1	2:1
N2	Span60	MP2	2	1	2:1
N3	Span60	MP3	2	1	2:1
N4	Span60	MP4	2	1	2:1
N5	Span60	-	-	-	2:1

**Table 2:** Nuclear abnormalities (NA) in human fibroblast (HDFa) cell cultures after applications with **MP1–4** for 24 h (125 μg/mL). Means followed by the same letter do not differ statistically (*p* > 0.05) from one another.

Treatments	Nuclear Abnormalities (NA)			
Isolates	Total MN	Total Lobbed	Total Notched	Mean NA/1000 Cells ± SD
(−) Ctrl	4	3	5	0.012 ± 0.002 ^a^
MP1	5	3	5	0.013 ± 0.001 ^a^
MP2	4	5	3	0.012 ± 0.002 ^a^
MP3	3	5	3	0.011 ± 0.001 ^a^
MP4	5	4	4	0.013 ± 0.003 ^a^

**Table 3:** The zeta potential energy, average particle size, polydispersity index, and percentage loading rates of drug-loaded formulations and the delivery system.

Group	ZP (mV)	d.nm	PDI	Drug Loading (%)
N1	−20.8	300	0.8	89
N2	−19.7	192	0.9	53
N3	−22.6	181	0.345	91
N4	−21.5	302	0.882	87
N5	−14.4	170	0.302	-

**Table 4:** Cell cycle distribution of SH-SY5Y cells treated with all-trans retinoic acid for 11 days as determined by flow cytometry.

	Cell Population (%)			
Group	G1 Phase	G2 Phase	S Phase	G2/G1
(−) Control	44.32 ± 1.42	12.64 ± 0.65	40.82 ± 3.12	2.22 ± 0.08
RA Treated	72.45 ± 3.78 *	5.34 ± 0.17 *	18.65 ± 3.23 *	3.56 ± 0.17

The values are expressed as mean ± standard deviation. The symbol (*) represents a statistically significant difference (*p* < 0.05) compared to the control.

**Table 5:** IC_50_ values (μg/mL) indicate the concentration required to achieve 50% cell viability for **MP1–4** compounds.

Compounds (MP)	IC_50_ (µg/mL)
MP1	5618.61
MP2	456.79
MP3	576.40
MP4	658.80

## Data Availability

Data are contained within the article.

## Supplementary Materials

The following supporting information can be downloaded at: https://www.mdpi.com/article/10.3390/pharmaceutics17020267/s1, Supplementary Data S1: Nuclear Magnetic Resonance Spectroscopy (NMR) Results of **MP1–4**.

## Abbreviations

The following abbreviations are used in this manuscript:

AD	Alzheimer’s disease
BBB	Blood–brain barrier
MTT	3-(4,5-Dimethylthiazol-2-yl)-2,5-Diphenyltetrazolium Bromide
MP1–4	Memantine derivatives 1 to 4
SEM	Scanning electron microscopy
UV	Ultraviolet
RA	Retinoic acid
Aß1-42	Amyloid beta 1-42 peptide
PDI	Polydispersity index
ZP	Zeta potential
SH-SY5Y	Human neuroblastoma cell line
DMEM	Dulbecco’s modified Eagle’s medium
PBS	Phosphate-buffered saline
