# Supplementary material for: Synthesis and Characterization of Memantine-Loaded Niosomes for Enhanced Alzheimer’s Disease Targeting
Source: Pharmaceutics. 2025 Feb 17;17(2):267. doi: 10.3390/pharmaceutics17020267 (PMC11860023; doi:10.3390/pharmaceutics17020267)
Supplement: Supplementary file 1 [file pharmaceutics-17-00267-s001.zip › pharmaceutics-3442305-supplementary.pdf]

Supplementary Data S1

Nuclear Magnetic Resonance Spectroscopy (NMR) Results are according to previously published ones (Cacciatore et al., 2017).

(MP1). Yield: 52%; Rf = 0.27, cyclohexane/Et_2O (1:1); 1H NMR (300 MHz, $CDCl_3$) δ: 0.84 (6H, s), 0.88 (6H, t, J = 6.9 Hz), 1.13–1.16 (2H, m), 1.19–1.39 (10H, m), 1.50–1.60 (4H, m), 1.65 (2H, s), 1.83 (3H, d, J = 3.0 Hz), 2.10–2.14 (1H, m), 5.10 (1H, br s); 13C NMR (75 MHz, $CDCl_3$) δ:14.2 (2 × CH_3), 20.9 (2 × CH_2), 30.0 (2 × CH_3), 30.1 (2 × C), 32.4, 35.5 (2 × CH_2), 40.3, 42.7 (2 ×CH_2), 47.7 (2 × CH_2), 48.6, 50.6, 53.4, 175.2. MS (ESI) m/z 328.30 $(M + Na)^+$. Anal. ($C_{20}H_{35}NO$) C, H, N.

(MP2). Yield: 57%; Rf = 0.56, cyclohexane/Et_2O (1:1); 1H NMR (300 MHz, $CDCl_3$) δ: 0.85 (6H, s), 1.14–1.36 (2H, m), 1.26–1.39 (4H, m), 1.63 (4H, s), 1.81 (2H, d, J = 2.7 Hz), 1.90–1.96 (2H, m), 2.06–2.14 (3H, m), 2.64 (2H, t, J = 7.5 Hz), 5.22 (1H, br s), 7.16–7.30 (5H, m); 13C NMR (75 MHz, $CDCl_3$) δ: 26.4, 27.2, 30.1 (2 × CH_3), 32.4, 33.4, 35.1, 36.8, 40.2, 42.7 (2 × CH_2), 47.6 (2 × CH_2), 50.6, 53.4, 125.9, 128.4 (2 ×CH), 128.5 (2 × CH), 141.6, 172.1. MS (ESI) m/z 348.27 $(M + Na)^+$. Anal. ($C_{22}H_{31}NO$) C, H, N.

(MP3). Yield: 51%; Rf = 0.52, cyclohexane/Et_2O (1:1); 1H NMR (300 MHz, $CDCl_3$) δ: 0.81 (6H, s), 0.90 (3H, t, J = 6.0 Hz), 1.11–1.13 (2H, m), 1.23–1.37 (4H, m), 1.53–1.63 (6H, m), 1.80 (2H, d, J = 3.0 Hz), 2.03 (2H, t, J =7.2 Hz), 2.08–2.11 (1H, m), 5.20 (1H, br s); 13C NMR (75 MHz, $CDCl_3$) δ: 13.7, 19.2, 30.0 (2 × CH_3), 30.1 (2 × C), 32.3, 39.6, 40.2, 42.7 (2 × CH_2), 47.6 (2 × CH_2), 50.6, 53.3, 172.3. MS (ESI) m/z 272.24 $(M + Na)^+$. Anal. ($C_{16}H_{27}NO$) C, H, N.

(MP4). Yield: 42%; Rf = 0.26, DCM; 1H NMR (300 MHz, DMSO-d_6) δ: 0.80 (6H, s), 1.09 (2H, s), 1.27 (4H, dd, J = 11.7 Hz, J = 14.1 Hz), 1.61 (4H, s), 1.79 (2H, s), 2.06 (1H, br s), 3.75 (6H, d, J = 4.2 Hz), 6.52 (1H, br s), 6.93 (1H, d, J =8.1 Hz), 7.03 (1H, d, J = 9.0 Hz), 7.08 (1H, s), 7.23 (1H, d, J = 15.9 Hz), 7.47 (1H, s); 13C NMR (75 MHz, DMSO-d_6) δ: 30.0, 30.5 (3 ×C), 32.3 (2 × CH_3), 42.8 (2 ×CH_2), 47.6 (2 × CH_2), 50.7, 52.8, 55.8, 55.9, 110.1, 112.1, 121.6, 122.0, 128.3, 138.1, 149.3, 150.3, 164.9. MS (ESI) m/z 392.27 $(M + Na)^+$. Anal. ($C_{23}H_{31}NO_3$) C, H, N.
